# Case Report and Literature Review of Cardiac Amyloidosis: A Not-So-Rare Cause of Heart Failure

**DOI:** 10.7759/cureus.33364

**Published:** 2023-01-04

**Authors:** Patrícia Baptista, Sofia Moura de Azevedo, André Alexandre, André Dias-Frias

**Affiliations:** 1 Internal Medicine, Centro Hospitalar Universitário do Porto, Porto, PRT; 2 Cardiology, Centro Hospitalar Universitário do Porto, Porto, PRT

**Keywords:** non-invasive multimodality diagnosis, al amyloidosis, transthyretin amyloidosis, cardiac amyloidosis, restrictive cardiomyopathy

## Abstract

Restrictive cardiomyopathy secondary to cardiac amyloidosis is an underdiagnosed cause of heart failure and it is associated with significant morbidity and mortality. The most common types of amyloidosis are light chain amyloidosis, transthyretin amyloidosis and secondary amyloidosis. We report the case of a 84-year-old man that presented with new onset signs and symptoms of heart failure. Multimodality imaging with echocardiogram and bone tracer cardiac scintigraphy along with biomarkers, monoclonal proteins analysis and genetic test allowed to diagnosed a wild-type transthyretin amyloidosis. We discuss the clinical and diagnostic features and review the current literature about cardiac amyloidosis. This paper aims to increase clinicians’ awareness of cardiac amyloidosis to promptly recognize, diagnose and treat it.

## Introduction

Restrictive cardiomyopathy secondary to cardiac amyloidosis is an underdiagnosed cause of heart failure [1,2]. It is associated with significant morbidity and mortality [1,2]. In a recent study performed by Mohan et al., the mean survival after the diagnosis of cardiac amyloidosis was 15 months, and the delayed diagnosis seems to be the major responsible factor for that [3]. Amyloidosis represents a group of systemic diseases that are characterized by organ deposition of misfolded protein fragments of several origins [2,4]. Cardiac amyloidosis refers to amyloid fibril deposition in the heart [2]. Amyloidosis is classified according to the precursor protein that forms the amyloid deposit [1,2,4,5]. The most common types of amyloidosis are light chain amyloidosis (AL), transthyretin amyloidosis (ATTR) and secondary amyloidosis (AA) [2,5]. Light chain amyloidosis results from deposition of misfolded immunoglobulin light chains caused by the proliferation of an abnormal clone of plasma cells [1,2,5]. Infiltration of soft tissue and small vessels can cause macroglossia and periorbital purpura (also called raccoon eyes), which are nearly pathognomonic [1,2]. In AL amyloidosis, patients typically present at age >40 years and the presence of cardiac amyloid infiltration is the main determinant of prognosis [2,5]. Transthyretin amyloidosis results from the misfolding and deposition of transthyretin [2,4,6]. Transthyretin is a stable tetramer that is produced by the liver, and it is responsible for the transport of thyroid hormone and retinol [2,4,6]. ATTR can be divided into two subtypes: wild-type amyloidosis (formerly known as senile amyloidosis) and hereditary amyloidosis [1,2,4-6]. Wild-type amyloidosis (wtATTR) typically presents at age >60 years and mainly involves the heart [2,5,6]. Soft tissue involvement is also frequently seen and it can manifest as bilateral carpal tunnel syndrome, spontaneous biceps tendon rupture and spinal stenosis [2,5,6]. Although uncommon, neuropathy can also be seen in up to 10% of patients [7]. The overall prevalence of wtATTR is unknown but recent literature suggests that up to 10% to 15% of older adults with heart failure may have undiagnosed wtATTR [2,7]. Hereditary transthyretin amyloidosis (hATTR) results from an inherited mutation in the transthyretin gene that leads to instability and misfolding of the tetrameric structure of transthyretin and subsequently deposition [2]. Besides the heart, it can also involve the peripheral and autonomic nervous systems [4,6]. Over 120 transthyretin mutations have been described, but the most common worldwide genetic variant is ATTRVal50Met [1,4-6]. Certain genotypes have been associated with a common deposition in certain organs, thus identifying the genetic mutation may assist to manage the patient [5]. AA amyloidosis is related to chronic inflammatory conditions and results from deposition of amyloid A-protein - an acute-phase reactant [4,5]. It frequently affects the kidneys but in late stages can affect other organs [4,5].

Cardiac amyloidosis presents with symptoms related to diastolic dysfunction and heart failure with preserved ejection function [1]. However, at later stages, patients can evolve with systolic dysfunction and heart failure with reduced ejection function [1]. Due to amyloid deposits in atrium and in the conduction system, patients can also present with atrial arrhythmias (frequently atrial fibrillation or flutter), sinus node dysfunction or advanced atrioventricular block and, less commonly, ventricular arrhythmia [1,2,8]. Low QRS voltage in electrocardiogram is the hallmark of cardiac amyloidosis, however this is more commonly seen in patients with AL [1,2]. Pseudoinfarct pattern with Q waves and loss of R waves in precordial lead is also possible [2]. Patients with cardiac amyloidosis often present with syncope or presyncope, which can result from conduction system disease or from hypotension caused by autonomic neuropathy or from excessive diuresis [2]. Additional cardiac manifestations may include effort angina, caused by small vessel disease, however epicardial coronary vessels at coronary angiography are usually normal [1].

## Case presentation

**Figure 1:**
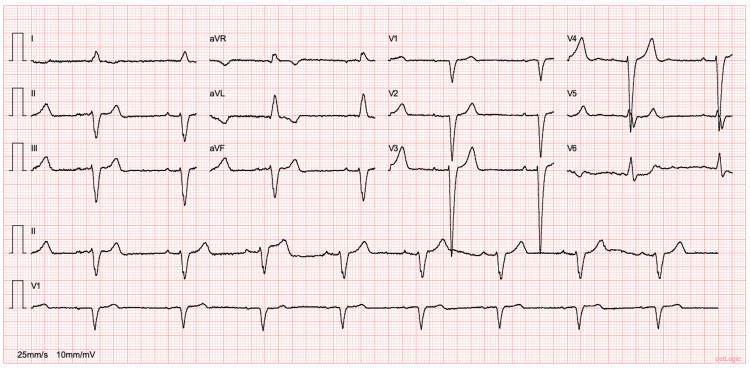
Electrocardiogram showing sinus rhythm (51/min), first degree atrioventricular block (PR interval of 218ms), and left bundle branch block.

An 84-year-old man, with history of dyslipidaemia and cervical myelopathy, was referred to an Internal Medicine appointment with a six-month history of leg swelling, dyspnea on exertion and orthopnea. The patient was also complaining of chest pain suggestive of effort angina and had already performed an exercise nuclear stress test that was negative for ischemia. The patient did not present any other clinical features, such as peripheral neuropathy, autonomic dysregulation, carpal tunnel syndrome, among other features suggestive of amyloidosis. At presentation, his electrocardiogram (ECG) showed first-degree atrioventricular block and left bundle branch block (Figure 1).

**Figure 2:**
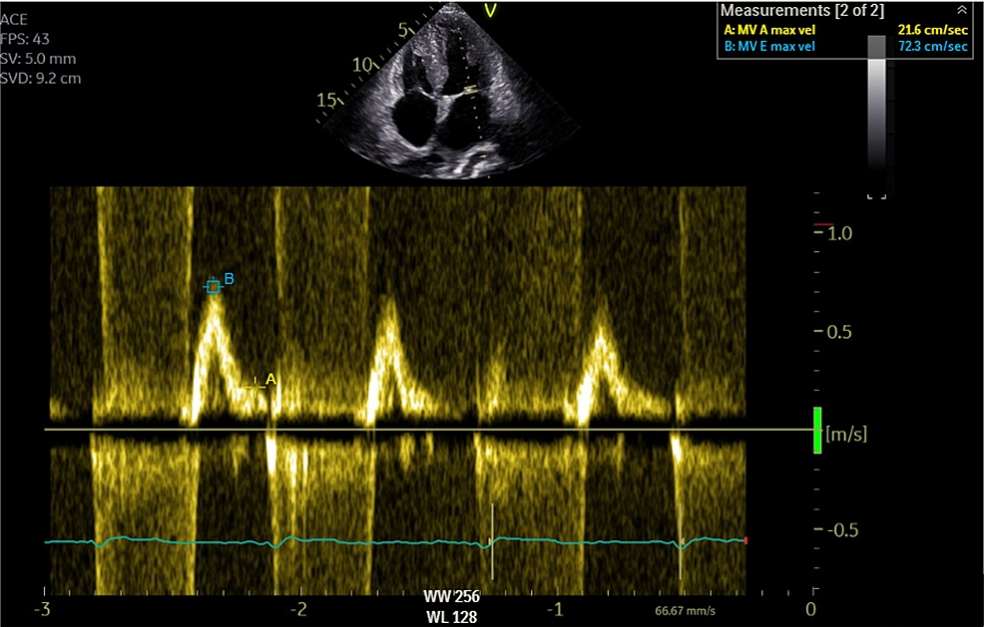
Echocardiography showing grade III diastolic dysfunction with an E/A ratio superior to 2. B = E wave; A = A wave

**Figure 3:**
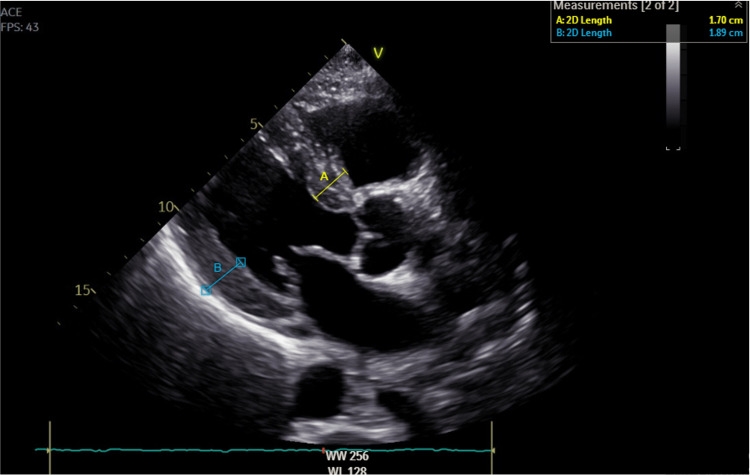
Parasternal long-axis view echocardiography revealing severe concentric left ventricular hypertrophy (posterior wall 19 mm), thickened interventricular septum (interventricular septum 17 mm), and atrioventricular valves. A = interventricular septum; B = posterior wall

Laboratory workup revealed normal complete blood count and renal and thyroid function tests. Pro-B-type natriuretic peptide (proBNP) was elevated at 4963 pg/mL (normal <300 pg/mL) and troponin T was also elevated to 0.103 ng/mL (normal <0.014 ng/mL). Coronary angiography showed normal coronary vessels. Transthoracic echocardiogram (TEE) revealed the presence of a restrictive/infiltrative cardiomyopathy, showing grade III diastolic dysfunction (Figure 2), concentric severe left ventricular hypertrophy and right ventricular hypertrophy, severe right and left atrial enlargement, thickened heart valves (Figure 3) and interatrial septum, moderate tricuspid regurgitation estimating a pulmonary artery systolic pressure of 57mmHg, moderately reduced left ventricular ejection fraction (38%) and mild pericardial effusion close the right atrium.

**Figure 4:**
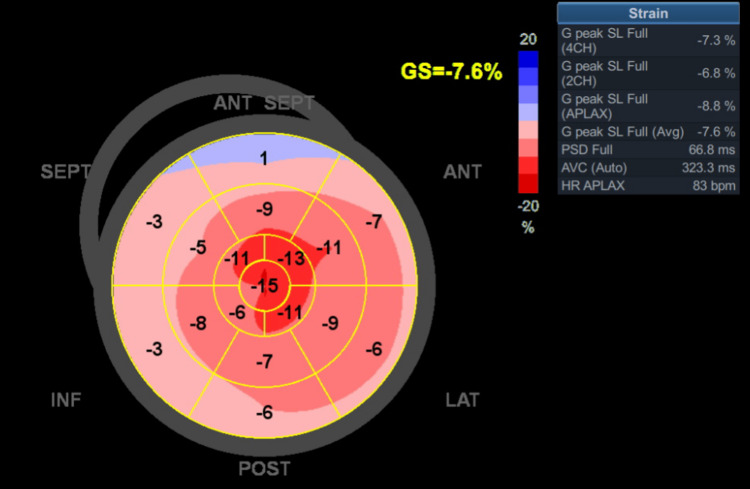
Bull’s eye graph of the left ventricle displacing the magnitude and homogeneity of longitudinal strain in color code map. It shows relative preservation of longitudinal strain at the apex resulting in a “cherry on top”/”apical sparing” pattern.

Global longitudinal strain (GLS) was severely reduced (-7.6%) with an “apical sparing” pattern suggestive of cardiac amyloidosis (Figure 4).

**Figure 5:**
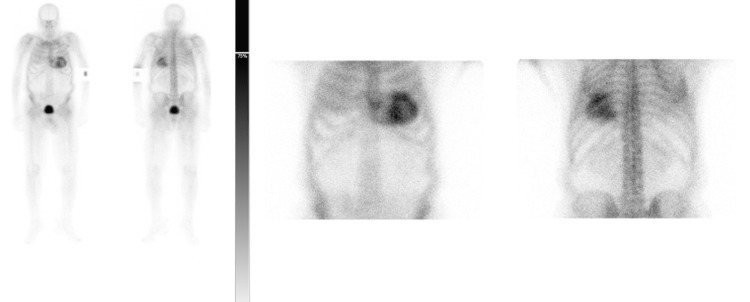
Scintigraphy with 99m-Technetium labeled 3,3-diphosphono-1,2-propano-dicarboxylic acid (DPD) showing myocardial uptake greater than bone with reduced/absent bone uptake – Perugini grade 3.

Therefore, the patient proceeded with diagnostic testing for amyloid cardiomyopathy. AL amyloidosis was excluded by serum and urine protein electrophoresis and immunofixation. Abdominal fat pad aspiration did not detect amyloid deposits. Genetic test did not show any transthyretin gene mutation. Radionuclide scintigraphy with 99m-Technetium labeled 3,3-diphosphono-1,2-propano-dicarboxylic acid (DPD) revealed a myocardial uptake greater than bone with reduced bone uptake - grade 3 (Figure 5).

Congestive symptoms were controlled with loop diuretics (furosemide 120mg per day) and he did not tolerate aldosterone antagonist due to hypotension. At the time of submission of this paper, the patient is under evaluation for treatment with tafamidis.

## Discussion

Echocardiogram is the main initial imaging test for the diagnosis of cardiac amyloidosis [1]. The main findings include moderate to severe left ventricular hypertrophy, biatrial enlargement, thickening of atrioventricular valves and interatrial septum, diastolic dysfunction with a restrictive filling pattern, and presence of mild pericardial effusion [1,2,9]. Left ventricular hypertrophy is typically symmetric in AL amyloidosis and asymmetric with predominantly septal hypertrophy in ATTR [2,5]. Greater increases in left and right ventricular mass and more systolic dysfunction are also more common in ATTR [2]. Reduction in global longitudinal strain with relative apical sparing pattern is a key feature of cardiac amyloidosis [1,2,9]. Cardiac magnetic resonance (CMR) is more sensitive and specific than echocardiogram, and identifies cardiac amyloidosis even in the early stages before the presence of left ventricular hypertrophy [2,5]. Initially, there may be a diffuse subendocardial pattern of late gadolinium enhancement, while in the late stages there is a transmural myocardial enhancement [1,2]. In cardiac amyloidosis, the deposition of amyloid fibrils occurs in the extracellular space, which produces an increase in extracellular volume [2]. Measurement of extracellular volume fraction in CMR can help to quantify the amount of cardiac amyloid [2]. Serum and urinary protein electrophoresis and immunofixation to identify monoclonal protein are mandatory in any patient with echocardiographic or CMR consistent with amyloidosis [1,2,6]. Ruling out monoclonal protein helps to support the diagnosis of ATTR [6]. Bone tracer cardiac scintigraphy using 99m technetium (Tc) labeled 3,3- diphosphono-1,2-propanodicarboxylic acid (DPD), pyrophosphate (PYP) or hydroxymthyleno diphophonate (HMDP) may help to identify and diagnose ATTR cardiac amyloidosis [2,9-11]. Then, it is applied the Perugini staging system based on visual scoring: grade 0, no cardiac uptake and normal bone uptake; grade 1, myocardial uptake in a lower degree than at bone level; grade 2, similar myocardial and bone uptake; grade 3, myocardial uptake greater than bone with reduced/absent bone uptake [2,10,11]. ATTR cardiac amyloidosis is especially avid for bone tracers so generally there is grade 2 or 3 uptake [2,11]. In contrast with AL amyloidosis, in which usually there is absent or grade 1 uptake [2,11]. Endomyocardial biopsy remains the gold standard for the diagnosis of cardiac amyloidosis since it shows amyloid deposits in cardiac tissue [1,2,5,6]. However, an abdominal fat pad aspiration is a better first choice procedure to do a histological diagnosis since it has a lower risk of adverse events [2,5,6]. However, in a study performed by Quarta et al. the sensitivity of abdominal fat pad aspiration in AL amyloidosis, hATTR and wtATTR was 84%, 45% and 15%, respectively [12]. The pattern of green birefringence under polarized light of the amyloid deposits with the Congo red staining is pathognomonic of amyloidosis [1]. The amyloid protein is identified using a laser microdissection with mass spectrometry [1]. However, according to Garcia-Pavia et al*.*, it is possible to establish the diagnosis of cardiac ATTR amyloidosis non-invasively providing that all of the following criteria are met: heart failure with an echocardiogram or cardiac magnetic resonance consistent with amyloidosis, grade 2 or 3 uptake on a radionuclide scintigraphy with 99m-Technetium labeled DPD or PYP, and absence of a detectable monoclonal protein [10]. For the remaining patients that do not fulfill all these criteria, it will be required an endomyocardial biopsy positive for amyloid deposits or extracardiac biopsy positive for amyloid, in addition to echocardiogram or CMR suggestive of cardiac amyloidosis [10]. As mentioned, non-invasive evaluation is the new backbone for the diagnosis of cardiac amyloidosis [13-15]. However no single imaging modality by itself is enough for the diagnosis of cardiac amyloidosis [13-15]. It is necessary an approach with multiple imaging modalities, including echocardiography, CMR and nuclear techniques, in addition to clinical features [13-15]. Multimodality imaging, mainly echocardiography and CMR, is also important for the prognosis and follow-up of these patients [13-15].

Natriuretic peptides and troponins are useful for risk stratification, and are also used to evaluate response to treatment and disease progression [16].

Regardless of patient age, genetic testing for the transthyretin gene is mandatory in all patients with ATTR since the results may have implications for family members and may also help to predict sites of organ involvement [6,7]. 

In the case of our patient it was possible to establish the diagnosis of ATTR wild-type amyloidosis with a combination of clinical features, echocardiography, bone tracer cardiac scintigraphy along monoclonal proteins analysis and genetic test.

Treatment of cardiac amyloidosis mainly encompasses measures to manage heart failure and other cardiac complications, but also disease-modifying therapy [1,2,4-9]. Initial treatment with loop diuretics and mineralocorticoid receptor blockers helps to improve symptoms related to congestion [1,2,4-9]. Angiotensin-converting-enzyme inhibitors, angiotensin receptor blockers and beta blockers are usually not well tolerated [2]. For atrial fibrillation, rhythm control with amiodarone or catheter ablation, along with anticoagulation, are the preferred strategies [8,9]. These patients usually do not tolerate beta blockers since the cardiac output is dependent on heart rate, and amyloid fibrils bind to digoxin which increases susceptibility to digitalis toxicity [2]. A permanent pacemaker may be required in patients with conduction system disease and implantable cardioverter-defibrillator may also be needed for secondary prevention of ventricular arrhythmias [10]. Specific disease-modifying treatment for AL includes chemotherapy followed by autologous stem cell transplantation [5]. For ATTR there are three groups of specific treatment: stabilizers, silencers and degraders [6-10]. Stabilizers, such as tafamidis, stabilize and prevent the misfolding of the native TTR tetramer, and can be used in wtATTR and hATTR [6-10]. Silencers, such as patisiran and inotersen, affect the production of TTR at the protein synthesis stage and therefore are used in patients with hATTR [6-10]. Degraders act to break down or remove amyloid deposits from the tissue, but they are still under active investigation [6-10]. Liver transplantation can be curative in selected patients with hATTR [2,7]. However, cardiac disease can progress even after liver transplantation [2].

## Conclusions

Amyloidosis is a systemic infiltrative disorder that can cause restrictive cardiomyopathy. Cardiac amyloidosis remains underdiagnosed, especially in elderly patients. Increased awareness of cardiac amyloidosis is essential to improve the understanding and achieve a timely and definitive diagnosis. Early diagnosis can provide more time to address disease progression and therefore prevent the patient from reaching an irreversible stage and a poor clinical outcome. This case also highlights the possibility of diagnosing TTR amyloidosis without any invasive procedure, namely a biopsy.
